# Non-adherence to preventive behaviours during the COVID-19 epidemic: findings from a community study

**DOI:** 10.1186/s12889-021-11506-0

**Published:** 2021-07-28

**Authors:** Róbert Urbán, Borbála Paksi, Ádám Miklósi, John B. Saunders, Zsolt Demetrovics

**Affiliations:** 1 Eötvös Loránd University, Institute of Psychology, Budapest, Hungary; 2 Eötvös Loránd University, Institute of Education, Budapest, Hungary; 3 Eötvös Loránd University, Institute of Biology, Budapest, Hungary; 4 the National Centre for Youth Substance Use Research, University of Queensland, Brisbane, Australia; 5 Centre of Excellence in Responsible Gaming, University of Gibraltar, Gibraltar, Gibraltar

**Keywords:** Adherence, Preventive behaviours, SARS-CoV-2, COVID-19, Hygienic behaviour

## Abstract

**Backgrounds:**

Preventive behaviours are an essential way to slow down and eliminate the transmission of SARS-CoV-2. The aim of this study was to estimate adherence to preventive behaviors and to identify whether any subgroups were not adopting these behaviours and for whom greater engagement in these approaches was indicated.

**Methods:**

In this cross-sectional study, we obtained data from a random sample of a panel representing men and women of adult age residing in Hungary (*N* = 5254). The survey included questions about the frequencies of preventive behaviours, perceived susceptibility and severity of COVID-19.

**Results:**

We found four factors of preventive behaviours: using physical barriers (mask and gloves), avoidance of close contacts, personal hygiene, and preparation. We identified two broadly adherent groups (36.8 and 45.3%) and two non-adherent groups (13.1 and 4.8%). Being male and being aged between 18 and 29 years were the strongest predictors of non-adherence. Concern about the severity of COVID-19 was a predictor of adherence.

**Conclusions:**

To ensure maximal adherence to preventive behaviours for COVID-19, additional strategies should focus on their adoption by men and young adults.

**Supplementary Information:**

The online version contains supplementary material available at 10.1186/s12889-021-11506-0.

## Introduction

The pandemic of COVID-19, caused by SARS-CoV-2, represents the most rapidly spreading infectious disease since the influenza pandemic of 1918–19. Responding effectively to the COVID-19 pandemic requires the collaborative efforts of health officials of all countries. It also necessitates people worldwide changing their individual behaviours in response to the public health imperative of infection control. Consequently social and behavioural sciences play central roles in coping with the challenges caused by the current COVID-19 and future pandemics [1, 2].

Possible routes of transmission include close contacts between people, contracting the virus though surface spread (via fomites), contracting it via large droplets (through coughing and sneezing), and airborne (aerosol) transmission especially in enclosed spaces [3]. Importantly, transmission of the SARS-CoV-2 is possible from infected people who are asymptomatic [4]. Preventive measures therefore need to be applied irrespective of apparent symptoms. Furthermore, the newly emerged variants of the SARS-CoV-2 impose further concerns and the need for public health programs, notwithstanding the roll-out of mass vaccination programs [5]. Infectivity is assessed by the basic reproduction number (R0) (i.e. without any intervention) and is estimated to be 3.32 [2.81–3.82] [6]. A key goal is to decrease this number to below 1.0 through coordinated prevention efforts which requires the engagement of members of the community and their adherence to these preventive approaches. Adherence to advice on preventive behaviours may be expected to reduce the spread of infection and give opportunity to the health care system to build up and maintain appropriate resources to treat severe cases [7]. The issue is whether and how this will be achieved in a sustained way.

Prevention of transmission of SARS-CoV-2, therefore, requires behaviour change that dramatically alters human interactions, independently of economic status, cultural heritage and geographical location. Countries facing the COVID-19 epidemic need to and most have implemented public health interventions and legislation to decrease the frequency of close personal contact by spatial distancing [8] and to promote physical or chemical barriers to virus transmission, through wearing face masks and gloves, and hand washing, respectively. A meta-analysis of studies investigating interventions to prevent influenza provided evidence of benefit from multiple preventive behaviours [9]. For example, hand washing in combination with face mask use reduces significantly influenza virus transmission [9]. Countries, however, differ in which protective measures are recommended. For example, the recommendation of wearing a face mask varies across countries [10, 11]. The degree of adherence necessary for effective prevention across populations is still not known, but we assume that consistent – every day or almost every day – use of preventive behaviours can significantly decrease the rate of viral transmission.

Understanding both distal (underlying) and proximal predictors of preventive behaviours may help predict different rates of viral submission in subgroups of the population. In most countries reporting death rates from COVID-19, these have been greater in men than women. In China and Italy deaths of men have been more than twice those of women. In New York City men constitute about 61% of patients who die [12, 13]. Men comprise 62.4% of COVID-19 deaths among those under 75 years of age in Hungary. Could it be that men are less likely to adopt preventive behaviours and more likely to become infected and die from the disease? Of course gender differences may be influenced by age, culture, other socioeconomic factors [14] or comorbidity status [12]. Preventive behaviours may play a role in the social disparities in SARS-CoV-2 infection, hospitalisation, and mortality [15].

In practical terms, the more immediate (proximal) predictors are of crucial importance. Health beliefs and other cognitive factors may explain different levels of adherence to preventive behaviours [16]. These include perceived susceptibility to, and perceived severity of the infection and its consequences. In the previous SARS pandemic, threat perception, perceived susceptibility and severity varied across countries [17]. However, in this study, the link between cognitive variables and infection control or preventive behaviours was not determined.

The present study had two aims. The first aim was to estimate the pattern of preventive behaviours and adherence and non-adherence to them. Adherence was defined as the person’s willingness to follow consistently those preventive behaviours which can decrease the chance of transmission of SARS-CoV-2. The second aim was to understand the role of distal factors such as gender, age, and socioeconomic status and proximal health beliefs in explaining the variance of preventive behaviours in the general population. It was hypothesised that men are less likely to adopt preventive behaviours than women. We also wished to examine particular preventive behaviours in young people and in those of lower socioeconomic status.

## Methods

### Participants and sampling method

In this cross-sectional study, participants were recruited from a panel of Hungarian adults (age > 18) using a computer-assisted web interview (CAWI) involving 74,500 currently active members as a sampling frame. A one-stage probabilistic random sampling method had been applied. The sample was stratified according to gender, age, education, and domicile, by over-representing the strata with low responsiveness to ensure that the final sample represents the Hungarian adult population according to the above characteristics. The sample size is planned to reduce sampling error below 1.5%. Five thousand two hundred fifty-four participants completed the survey. To compensate for sample disproportions, an iteration weighting by layer categories was used. The socio-demographic features of the sample are presented in Table 1.

**Table 1:** Descriptive statistics and sex difference

	Total	Males	Females	t-test/χ^**2**^	Effect size - Cohen d
*Demographic variables*^*a*^					
Sex, *Females N (%)*	3247 (61.8)				
Age, *Mean (SD)*	53.8 (14.4)	56.38 (14.19)	52.25 (14.37)	10.15***	0.29
Education:					
Less than high school *N (%)*	683 (13.0)	319 (15.9)	364 (11.2)	42.1***	0.09^#^
High school *N (%)*	2374 (45.2)	807 (40.2)	1567 (48.3)		
College or University *N (%)*	2197 (41.8)	881 (43.90)	1316 (40.50)		
Settlement type					
Budapest (capital)	952 (18.1)	372 (18.5)	514 (15.8)	14.2**	0.05^#^
County-town	940 (17.9)	729 (36.3)	1322 (40.7)		
Other city	1787 (34.0)	596 (29.7)	889 (27.4)		
Village and smaller settlements	1575 (30.0)	310 (15.4)	522 (16.1)		
Having any chronic disease *N (%)*^b^	1788 (34.0)	720 (36.6)	1068 (33.4)	5.34*	0.06
Preventive behaviours^c^	Always *N (%)*	Mean (SD)	Mean (SD)		
Washing hands when arrived home	4583 (88.7)	4.70 (0.79)	4.88 (0.50)	10.12***	0.29
Avoiding contacts while greetings (hugging, kissing)	4334 (83.5)	4.68 (0.84)	4.74 (0.74)	2.73**	0.08
Avoiding handshake	4061 (78.4)	4.49 (0.96)	4.77 (0.70)	13.60***	0.39
Avoiding meeting with group of people	3932 (75.8)	4.41 (1.10)	4.68 (0.80)	10.07***	0.29
Avoiding public transportation	3691 (71.7)	4.35 (1.22)	4.44 (1.13)	2.63**	0.08
Washing hand when outside home	3581 (69.3)	4.29 (1.10)	4.58 (0.92)	10.30***	0.30
Staying home, and leaving home only when it is necessary	3458 (66.8)	4.29 (1.06)	4.62 (0.78)	12.67***	0.35
Avoiding people who have high risk of complication (old people)	3343 (64.4)	4.31 (1.07)	4.50 (0.92)	6.93***	0.19
Taking vitamin supplementation	2873 (55.0)	3.82 (1.44)	4.13 (1.31)	8.21***	0.23
Using hand sanitizer	2285 (44.4)	2.89 (1.73)	3.71 (1.65)	17.20***	0.49
Keeping at least 2 m distance from others	2043 (39.6)	3.86 (1.16)	4.14 (1.00)	9.41***	0.27
Avoiding self-touching (face, mouth, eyes)	2067 (39.3)	3.76 (1.22)	4.16 (1.02)	12.83***	0.37
Wearing face mask	1043 (20.3)	2.42 (1.56)	2.73 (1.65)	6.67***	0.19
Wearing protective gloves	1021 (19.9)	2.37 (1.51)	2.79 (1.64)	9.20***	0.26
Storing food	540 (10.3)	2.44 (1.34)	2.64 (1.40)	5.11***	0.15
Having someone in the household who are at risk of developing severe condition^d^	3205 (60.0)	1262 (62.9)	1862 (57.3)	15.76***	0.11
Number of days left home for work during the last 7 days^e^	2.88 (2.40)	3.32 (2.56)	2.62 (2.26)	10.34***	0.30
Number of days left home for shopping, pharmacy during the last 7 days^e^	3.02 (1.81)	3.37 (1.97)	2.80 (1.67)	11.17***	0.32
Number of days left home for other reasons during the last 7 days^e^	1.83 (1.66)	2.01 (1.80)	1.72 (1.55)	6.14***	0.18

*Note*: Data are presented without the sampling weights. ^#^: Phi coefficient. *** *p* < 0.001; ** *p* < 0.01. Boldfaced t-values are still significant after the Bonferroni correction of multiple testing (*p* < .002). ^a^: No missing value. ^b^: Number of missing values is 89 (1.7%). ^c^: Numbers of missing or no responses were between 28 (0.5%) and 114 (2.1%). ^d^: Number of missing values is 48 (0.9%). ^e^: Numbers of missing values are between 12 (0.2) and 68 (1.3)

Participation of this study was anonymous and voluntary. The participants were requested to construct a code which made it possible to contact them later for a possible follow-up data-collection. This study was performed in accordance with Helsinki declaration and approved by the Institutional Review Board of Eötvös Loránd University, Budapest, Hungary (no: 2020/134). Informed consent was obtained from all participants.

### Study setting

**Fig. 1:**
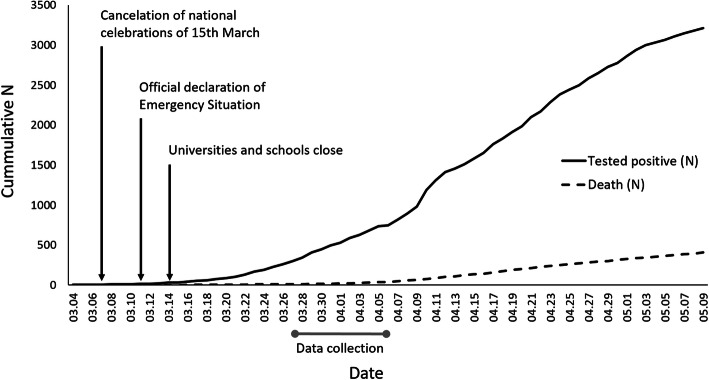
SARS-CoV-2 pandemic context of present data collection

Before the time of data collection, which began on 27 March 2020 and ended on 6 April 2020, Hungary had experienced the onset of SARS-CoV-2 transmission and COVID-19 disease (see Fig. 1). However, throughout the study, Hungary had a relatively low number of infections and a low death rate, which was seen predominantly in those over 65 years of age. The Hungarian Government declared a state of emergency on 11 March and started curfew restrictions on 28 March, which on 9 April were extended for an unlimited time.

### Measures

#### Outcomes

##### Preventive behaviours

Fifteen statements were constructed to embrace a wide range of preventive behaviours including:

- wearing disposable gloves,
- wearing a face mask,
- using hand sanitiser,
- hand washing,
- avoidance of facial self-touching,
- keeping at least 2 m distance from another person,
- avoiding public transportation,
- avoiding close contacts, and
- avoiding meetings.

The full list of questions is presented in Table 1. The response options were provided such as never (1), sometimes (2), 50% of cases or times (3), almost always (4) and always (5).

#### Adherence to “stay at home” message

Three questions asked the participants’ behaviour during the past 7 days: how many days they left their home (1) for work, (2) for buying food and visiting a pharmacy; (3) for other reasons. The respondents had to indicate the number of days over the past 7 days.

#### Predictors

##### Perceived susceptibility

Six questions enquired about the likelihood of (a) contracting the virus without knowing it; (b) contracting the virus and having no or mild symptoms; (c) mild suffering from the infection; (d) contracting the infection and being placed in quarantine; (e) getting hospitalised due to the infection; (f) getting into the intensive care unit in a hospital. Responses were recorded on a 5-point from unlikely (1) to very likely (5). Based on the item correlations, the principal component analysis revealed two factors, namely (i) likelihood of being asymptomatic or having mild symptoms and (ii) likelihood of severe conditions due to coronavirus (explained variance = 76.9%). In further analyses, we used these two factors: the perceived likelihood of being asymptomatic or having mild symptoms (Cronbach α = .74), and the perceived likelihood of a severe condition (Cronbach α = .88). Two scores summated the mean of the relevant items.

##### Perceived severity

Four questions were constructed to measure the perceived impact of novel coronavirus infection on individual and family health. Responses were recorded on a 5-point Likert type scale from not at all (1) to very much (5). The principal component analysis revealed one component (explained variance = 76.9%). The scale score was calculated with the mean of the responses (Cronbach α = 0.85).

##### Cues to action

Two questions assessed cues to preventive actions: (1) in the household, how many people are vulnerable to severe coronavirus infection; (2) if the participant suffers from any chronic health condition.

### Statistical analysis

Beyond the descriptive statistics, the following analyses were determined by the data analysis strategy:

1. An exploratory factor analysis (EFA) was carried out on the preventive behaviours in order to identify the main factors of these behaviours. The responses were used as ordinal indicators with GEOMIN (an oblique-type) rotation. Eigenvalues greater than 1, inspection of the scree plot, and interpretability of the factor solution were used in determining the number of extracted factors. Based on the factor solution, scale scores were calculated by averaging the items belonging to each factor.
2. We aimed to identify groups of people exhibiting similar patterns of preventive behaviour to detect the proportion of people who are less prone to COVID-19 due to consistent use of preventive measures. Latent profile analysis was therefore performed with preventive behaviour factors as observed indicators. The latent profile analysis [18] is a latent variable analysis with a categorical latent variable – in this case adherence types – and continuous manifest indicators such as preventive behaviour factors. In the process of determining the number of latent classes, the Bayesian information criteria was used, alongside the interpretability of clusters. Furthermore, the Lo-Mendell-Rubin Adjusted Likelihood Ratio Test was also used. This compares the estimated model with a model having one less class than the estimated model. A low *p* value (<.05) indicates that the model with one less class is rejected in favor of the estimated model.
3. We compared the latent classes using the most likely class membership across distal socio-demographic variables and the proximal variables such as health beliefs, cues to action and finally we also used the behavioural indicators of the degree of following the “stay at home” message to validate concurrently our classes.
4. Finally, we merged the non-adherent groups and the adherent groups and performed two-step hierarchical binary logistic regression: in the first step we entered the distal explanatory variables and then in the second step we also add the proximal explanatory variables.

All analyses were performed with SPSS 22 (IBM SPSS Inc., Chicago, Illinois) and MPlus 8.0 program [19]. The data that support the findings of this study and the used Mplus syntax files are available from the corresponding author upon reasonable request.

## Results

### Descriptive statistics

The descriptive statistics are presented in Table 1*.* The most frequently used preventive behaviours were hand washing and avoiding different types of close contact with other people. Consistent gender differences were detected: males performed preventive behaviours less often than females (Table 1) with effect sizes mostly in the medium range. Men left home for specified and unspecified reasons more frequently than women.

### Measuring preventive behaviours and identifying adherence types

We performed exploratory factor analyses to explain the covariances among the preventive behaviours. The detailed description of the exploratory factor analysis (EFA) and factor loadings are to be found in the supplement of the present paper. Our analyses yielded four factors: (1) using a *physical barrier* such as wearing a face mask and protective gloves; (2) *avoidance of close contacts* such as avoiding hugging/kissing, shaking hands, avoidance of meetings, and avoidance of people who are at high risk; (3) *personal hygienic behaviours* such as hand washing, hand sanitisers, avoidance of self-touching, and keeping 2 m distance from other people; (4) *preparation* including vitamin intake and storing food. The correlations among these factors ranged between 0.30 and 0.55 (Table 1).

**Fig. 2:**
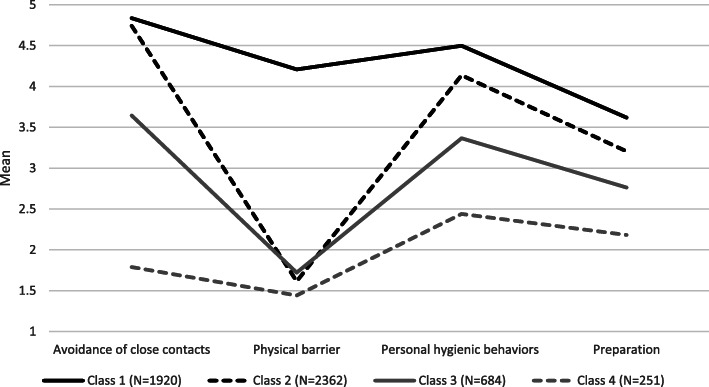
Latent profile analysis: Classification of adherence to protective behaviours. Note: The sizes of latent classes are estimated with the use of sampling weights. Unweighted frequencies were: Class 1 (Full adherence): *N* = 2034; Class 2 (Partial adherence): *N* = 2519; Class 3 (Limited adherence): *N* = 509; Class 4 (Non-adherent): *N* = 174. The range of means is between 1 (never) and 5 (always)

Based on the EFA, the means of items within their respective factors were calculated and used as observed indicators in the latent profile analysis. Single- to 5-class solutions were estimated. The decision regarding the number of classes is documented in the supplemental Table 2. A four-class solution was retained (Fig. 2). According to our operationalisation, adherence to preventive behaviours is defined as performing the behaviour almost always or always, which required the range of mean score to be between 4 and 5. Below this value we regarded as limited adherence or non-adherence. The most adherent class (Class 1 – *Full adherence*) comprised 36.8% of the sample. This class showed consistent use of all four preventive behaviours. The averaged ratings fell between always and almost always in spatial distancing, physical barriers and personal hygienic behaviours. The second most adherent class (Class 2 – *Partial adherence*) comprised 45.3% of people. The members of this group used spatial distancing and personal hygienic behaviours always or almost always, but they used physical barriers (mask and gloves) less frequently. The third class (Class 3 – *Limited adherence*) represented 13.1% of the participants. This group is characterised by the use of spatial distancing and personal hygienic behaviours around half of all occasions; however, this group hardly uses physical barriers. Finally, the fourth class (Class 4 – *Non-adherence*) represented 4.8% of people. This group did not follow the recommended actions regularly, and they mentioned only the occasional use of personal hygienic behaviours to prevent coronavirus infection.

### Characteristics and validation of the adherence typology

The latent classes were compared along distal demographic and proximal health belief variables (Table 2). The *full adherence* group (Class 1) differed in most characteristics from the other three groups, being characterised by higher mean age, higher proportion of women, reporting stronger perceived severity of COVID-19, higher perceived susceptibility to a serious condition, and higher rates of having someone who is vulnerable to the serious impact of COVID-19, and having at least one chronic health condition. The *partial adherence* group (Class 2) differed in most of the above characteristics from the full adherence group, however in some characteristics (i.e., proportion of women, perceived severity of COVID-19) showed an in-between position compared to the other two groups. The *limited adherence* (Class 3) and the *non-adherent* (Class 4) groups are similar in age and gender distribution by representing younger age groups and higher proportions of men compared to the other two adherent groups. These groups also reported leaving home for a reason other than work and shopping more frequently than the two more adherent groups.

**Table 2:** Comparisons of latent classes of preventive behaviours

	Class 1 – Full adherence ***N*** = 2034	Class 2 – Partial adherence ***N*** = 2519	Class 3 – Limited adherence ***N*** = 509	Class 4 – Non-adherence ***N*** = 174	F/χ^**2**^
Class indicator variables					
Avoidance of close contacts *Mean (SD)* *95% CI*	4.84_a_	4.74_b_	3.62_c_	1.78_d_	7166.5***
	(0.26)	(0.28)	(0.41)	(0.59)	
	4.83–4.85	4.73–4.75	3.58–3.66	1.70–1.88	
Physical barrier *Mean (SD)* *95% CI*	4.23_a_	1.60b_c_	1.70_b_	1.44_c_	5197.3***
	(0.72)	(0.68)	(0.94)	(0.90)	
	4.20–4.26	1.57–1.62	1.62–1.79	1.31–1.58	
Personal hygienic behaviours *Mean (SD)* *95% CI*	4.51_a_	4.13_b_	3.34_c_	2.43_d_	923.4***
	(0.47)	(0.66)	(0.82)	(1.06)	
	4.49–4.53	4.10–4.15	3.27–3.41	2.27–2.5	
Preparation *Mean (SD)* *95% CI*	3.66_a_	3.17_b_	2.77_c_	2.18_d_	205.6***
	(0.97)	(1.07)	(1.09)	(1.08)	
	3.61–3.70	3.13–3.21	2.68–2.87	2.02–2.34	
Distal and proximal variables					
Age, *Mean (SD)*	57.07_a_	52.33_b_	49.88_c_	49.45_bc_	63.5***
	(13.93)	(14.08)	(14.80)	(16.60)	
Sex, females N (%)	1422_a_	1521_b_	221_c_	72_c_	163.3***
	(69.9)	(60.4)	(43.4)_c_	(41.1)	
*Education*					
Less than high school *N (%)*	283_a_	285_b_	84_a_	26_ab_	18.7**
	(13.9)	(11.3)	(16.5)	(14.9)	
High school *N (%)*	933_a_	1124_a_	232_a_	77_a_	
	(45.9)	(44.6)	(45.6)	(44.0)	
College or University *N (%)*	818_a_	1110_b_	193_a_	72_ab_	
	(40.2)	(44.1)	(37.9)	(41.1)	
*Settlement*					
Budapest N (%)	355	421	77	31	1.7
	(17.5)	(16.7)	(15.1)	(17.7)	
*Health beliefs*					
Perceived susceptibility of severe condition *Mean (SD)*	2.88_a_	2.65_b_	2.67_b_	2.47_b_	20.1***
	(1.17)	(1.03)	(1.03)	(1.16)	
Perceived susceptibility of asymptomatic or mild condition *Mean (SD)*	3.22_a_	3.37_b_	3.38_b_	3.20_a_	11.9***
	(0.98)	(0.89)	(0.89)	(1.14)	
Perceived severity *Mean (SD)*	4.10_a_	3.70_b_	3.53_c_	3.24_d_	142.6***
	(0.77)	(0.83)	(0.86)	(1.15)	
*Cues to action*					
Having vulnerable family members in the household. N (%)	1295_a_	1421_b_	301_b_	96_b_	68.2***
	(79.3%)	(67.3)	(71.5)	(67.1)	
Having a chronic disease N (%)	816_a_	763_b_	149_b_	56_b_	56.6***
	(40.9)	(30.7)	(30.1)	(32.4)	
Concurrent validation: Behavioural indicators during the past 7 days					
Number of days left home for work *Mean (SD)*	2.49_a_	3.04_b_	3.53_c_	3.34_b_	37.1***
	(2.23)	(2.41)	(2.63)	(2.70)	
Number of days left home for shopping, pharmacy *Mean (SD)*	2.65_a_	3.13_b_	3.74_c_	3.60_b_	66.6***
	(1.61)	(1.77)	(2.11)	(2.51)	
Number of days left home for other reasons	1.58_a_	1.90_b_	2.30_c_	2.37_c_	37.6***
	(1.41)	(1.70)	(1.95)	(2.21)	

*Note:* Games-Howell post hoc test was used for pairwise comparisons of means. Proportions are compared with z-test. *: *p* < 0.05; ***: *p* < 0.001. *CI* confidence interval. Values in the same row not sharing the same subscript are significantly different at *p* < 0.05. Analyses are performed with the unweighted dataset

### Predictors of non-adherence to prevent SARS-CoV-2 transmission

Based on our latent class analyses, we merged the two adherent groups (Classes 1 and 2) and the two limited or non-adherent groups (Classes 3 and 4) to perform a hierarchical binary logistic regression analysis in order to predict non-adherence with the distal socio-demographic, and proximal health beliefs variables. We entered the variables in two blocks (see Table 3).

**Table 3:** Predictors of non-adherence to preventive behaviours: a binary logistic regression analysis

	***Model 1***		***Model 2***	
	Odds Ratio	95% CI	Odds Ratio	95% CI
Sex				
Males	2.53***	[2.15–2.97]	2.37***	[2.00–2.80]
Females	Ref.		Ref.	
Age				
18–29 years	4.30***	[3.30–5.61]	2.87***	[2.14–3.86]
30–49 years	1.54**	[1.21–1.97]	1.25	[0.95–1.63]
50–64 years	1.14	[0.88–1.48]	1.04	[0.79–1.37]
65≤	Ref.		Ref.	
Education				
Less than high school	1.41**	[1.12–1.77]	1.42***	[1.12–1.81]
High school	1.17	[0.92–1.50]	1.17	[0.91–1.50]
College or University	Ref.		Ref.	
Settlement types				
Budapest (capital)	Ref.		Ref.	
County-town	1.22	[0.96-1.54]	1.18	[0.89–1.55]
Other city	1.26*	[1.00–1.58]	1.50**	[1.18–1.90]
Village and smaller settlements	1.00	[0.77–1.30]	1.31*	[1.02–1.68]
Having vulnerable family members in the household				
No	1.28**	[1.10–1.49]	1.11	[0.94–1.30]
Yes	Ref.		Ref.	
Having a chronic illness				
No	1.06	[0.88–1.27]	0.96	[0.91–1.08]
Yes	Ref.		Ref.	
Perceived susceptibility of asymptomatic or mild condition	–		0.95	[0.87–1.03]
Perceived susceptibility of severe condition	–		0.99	[0.91–1.08]
Perceived severity	–		0.67***	[0.60–0.75]

Note: *N* = 5152. (missing is *N* = 102). *CI* confidence intervals. *:*p* < 0.05; **:*p* < 0.01; ***:*p* < 0.001. Boldfaced coefficients are significant at least at *p* < 0.05. Binary outcome variable: 0: adherent and 1: non-adherent. Analyses are performed with the unweighted dataset

In the first model, only the distal variables were entered. Non-adherence was more likely among males, in younger age groups and in those with less than high school education (see Table 3). Males had 2.5 times higher odds of not following the preventive behaviours, irrespective of the age groups (see Supplementary Table 3). Age between 18 and 29 was found to have 4.3 times higher odds of not performing the preventive behaviours compared to the older group. Having less than high school education increased the odds of non-adherence by 41%. Finally, we also tested that lack of someone in the household who is at risk of developing severe COVID-19 also increased the odds of non-adherence compared to those who have such relatives.

In Model 2, we also added the proximal variables resulting that only perceived severity decreased the odds of being non-adherent. Observing the decrease of the odds ratio of younger age in Model 2 from Model 1 implies that perceived severity may explain the link between age and non-adherence partly.

## Discussion

It is encouraging that most participants reported adhering to the main preventive behaviours recommended by the public health authorities. However, it is concerning that men throughout the age ranges were less likely to adopt the recommended measures than women, and this applied to all four preventive approaches. Young people were less likely to adopt preventive approaches than those aged 30 years or more, which was related in part to lower perceived susceptibility to a severe COVID-19 illness.

The SARS-CoV-2 incidence rate has remained relatively low in Hungary probably due to forthright decisions to implement broadly-based public health approaches at an early stage. These include measures aimed at spatial distancing and adopting hygienic approaches that were introduced at an early stage of the pandemic. Based on our results, a large proportion of the population adhered to the recommendation of avoiding close contacts and personal hygienic behaviours to control viral transmission. However, not all behaviours were followed equally: using face masks and protective gloves were less common and more variable among people who were inclined to follow all the other protective measures.

In our study, nearly 18% of the sample was identified as non-adherent or have limited adherence to the public health recommendations. Non-adherence rates in lifestyle-related behaviour change advice are around 30% [20]. The non-adherence rates reported in the present study are therefore lower but are more concerning because of the implications for imminent morbidity and mortality. Nearly 20% of persons in the present study reported a low level of adherence to spatial distancing, face mask use and/or taking personal hygienic precautions; thus, they have an increased risk of contracting and transmitting the SARS-CoV-2 virus and other respiratory infections in the future. At the time when countries try to relax the tight control over virus transmission, it becomes even more important to know the proportion of non-adherent individuals and the predictors of response to preventive advice. The present data were collected during the first wave of the pandemic in Hungary; therefore, it represents the collective response to the new challenge. Therefore, our results may be informative in future outbreaks of variant coronaviruses and similar threats.

Several countries have experienced higher rates of severe COVID-19 illness and deaths among men compared with women [12, 13, 21]. This should be compared with the data from the present study of male gender being another important predictor of non-adherence. An increased risk of death among males was documented in relation to the previous SARS-virus induced acute respiratory syndrome, in which males had 66% higher risk of dying than females [22]. Several hypotheses have been proposed to explain this gender difference, including different rates of smoking [23, 24]. Our data highlight the gender difference in adherence to preventive behaviours. Since we do not have data about the smoking status of our participants we cannot control for the covariance between smoking and non-adherent behaviours. Previous studies also supported the gender disparities in handwashing behaviour and knowledge regarding personal hygienic behaviours [25, 26]. Our results regarding the gendered preventive behaviours are in accordance with other studies from different countries that documented gender disparities in adherence behaviours (see for a brief review [27]. However, further research is necessary to disentangle the multiple behavioural mechanisms explaining why males seem to be more vulnerable to respiratory infections. We also need research on the gendered meaning of preventive behaviours that would explain the lower rate of preventive behaviours among men [28]. Lower rate of adherence, lower level of self-care and/or lower level of perceived threat among males may be important factors explaining the higher rate of general mortality among men compared to women [29].

Being of a younger age is an important predictor of non-adherence to preventive behaviours [27]. Frequent communication regarding the SARS-CoV-2 virus stressed the fact that COVID-19 threatens mainly the older population. This could lead to a false safety message to younger people [30], though 1% of hospitalisation due to COVID-19 in China was among those aged 20–29 years, and 3% from the age group of 30–39 years old [31]. Based on nine relevant health indicators, approximately one-third of young people are medically vulnerable to severe COVID-19 illness, at least in the U.S. [32]. Lower adherence among younger people may be associated with their perceived lower vulnerability and their tendencies to make impulsive decisions, take more risks to gain social and emotional stimulation (such as social gatherings), and underestimate the long-term consequences of their behaviour [33]. Our study demonstrated different rates of preventive behaviours. The variance of the use of physical barriers may be due to several factors including (1) the lack of practice of face mask use; (2) the social meaning of face masks and gloves in Europe; and (3) messages from the WHO and the country’s officials regarding the use of face masks and protective gloves (WHO, 2020). However, previous experience with prevention of influenza emphasised the use of face mask with hand hygienic measures [9]. It is important to note that face mask use did not decrease the frequency of the use of other preventive techniques, therefore it did not increase illusory safety. It can be recommended without the serious side effect of neglecting other infection control means. Furthermore, recent evidence supports the idea that face mask can protect from the transmission of viral RNA [34].

Our study is unique since we also focused on patterns of adherence behaviours. Previous studies focused mainly on predicting or explaining specific preventive behaviours; however, in the case of the complex set of preventive behaviours which can prevent viral transmission, the person-oriented type of analysis focusing on the pattern of behaviours has the specific advantage in identifying subgroups of the population sharing similar characteristics. Thus, in our study, we identified subgroups with different levels of adherence. The understanding of the covariates of these subgroups informs the prevention experts how to tailor health promotion programs. This study is also different from those studies using online convenience samples because we applied a probabilistic random sampling method, which enabled us to have a sample that proportionally represents the Hungarian adult population. Furthermore, this sampling method significantly reduces the selection bias.

Our study is not without limitations. The cross-sectional design does not allow definitive conclusions about causation, and self-reported data may be distorted by social desirability bias. An independent direct observational study could validate our findings. Noteworthy, independent observational studies of face mask use behaviours [35, 36] and handwashing behaviours [25, 26] validated our findings regarding the gender difference in preventive behaviours. However, in the present pandemic there is a compelling need for timeliness of studies. Understanding adherence to public health recommendations will help to decrease the likelihood of SARS-CoV-2 transmission and potentially the severity of the COVID-19 illness, and should help us in the future to prevent and contain influenza and other still unknown viral pandemics. Everyone, irrespective of their particular risk of these infections, can contribute to the health of the community as a whole. The findings from the present study emphasise that developing ways of engaging men, young people and those of low socioeconomic status in adopting preventive behaviours and emphasising the severity of the illness is vital not only for optimal prevention of SARS-CoV-2 transmission now and in the future, but also for effective control of related respiratory infections.

## Supplementary Information

**Additional file 1: Supplementary Table 1:** Exploratory factor analysis of SARS-CoV-2-related preventive behaviours. **Supplementary Table 2**. Fit indices of different latent class solution. **Supplementary Table 3.** Sex as a predictor of non-adherence in different age groups.

## Data Availability

The dataset used and analysed in this study is available from the corresponding author on reasonable request.
