# Supplementary material for: Non-adherence to preventive behaviours during the COVID-19 epidemic: findings from a community study
Source: BMC Public Health. 2021 Jul 28;21:1462. doi: 10.1186/s12889-021-11506-0 (PMC8316539; doi:10.1186/s12889-021-11506-0)
Supplement: Supplementary file 1 — Additional file 1: Supplementary Table 1: Exploratory factor analysis of SARS-CoV-2-related preventive behaviours. Supplementary Table 2. Fit indices of different latent class solution. Supplementary Table 3. Sex as a predictor of non-adherence in different age groups. [file 12889_2021_11506_MOESM1_ESM.docx]

Non-adherence to preventive behaviours during the COVID-19 epidemic: findings from a community study

Róbert Urbán, Borbála Paksi, Ádám Miklósi, John B. Saunders, & Zsolt Demetrovics^(5)^

**Supplementary Table 1:** Exploratory factor analysis of SARS-CoV-2-related preventive behaviours.

|  | **Physical barrier** | **Avoidance of close contacts** | **Personal hygienic behaviours** | **Prepa-ration** |  |
| --- | --- | --- | --- | --- | --- |
|  | **Standardized factor loadings** | | | | **Comm.** |
| Wearing protective gloves | **0.842** | 0.032 | 0.013 | 0.014 | 0.747 |
| Wearing face mask | **0.873** | 0.005 | 0.003 | 0.030 | 0.784 |
| Avoiding contacts while greetings (hugging, kissing) | -0.027 | **0.969** | -0.058 | -0.005 | 0.572 |
| Avoiding handshake | 0.003 | **0.865** | 0.019 | 0.052 | 0.802 |
| Avoiding meeting with groups of people | 0.047 | **0.775** | 0.008 | 0.046 | 0.665 |
| Staying home, and leaving home only when it is necessary | 0.091 | **0.612** | 0.102 | 0.025 | 0.524 |
| Avoiding people who have high risk of complication (old people) | -0.026 | **0.610** | 0.068 | 0.084 | 0.454 |
| Washing hand when outside home | -0.054 | -0.005 | **0.775** | 0.116 | 0.633 |
| Washing hand when arrived home | -0.069 | 0.248 | **0.771** | 0.021 | 0.833 |
| Avoiding self-touching (face, mouth, eyes) | 0.106 | 0.203 | **0.582** | -0.016 | 0.572 |
| Using hand sanitizer | 0.148 | -0.166 | **0.456** | 0.281 | 0.354 |
| Keeping at least 2 meters distance from others# | 0.149 | **0.390** | **0.455** | -0.117 | 0.606 |
| Avoiding public transportation# | 0.007 | **0.362** | **0.313** | -0.031 | 0.344 |
| Taking vitamin supplementation | -0.019 | 0.108 | 0.087 | **0.583** | 0.433 |
| Storing food | 0.079 | 0.081 | -0.097 | **0.474** | 0.260 |
| Eigenvalues | 6.593 | 1.485 | 1.232 | 1.097 |  |
| Factor correlations* | | | | |  |
| Avoidance of close contacts | 0.369 |  |  |  |  |
| Personal hygienic behaviours | 0.358 | 0.546 |  |  |  |
| Preparation | 0.311 | 0.340 | 0.301 |  |  |
| Cronbach α | 0.68 | 0.81 | 0.70 | 0.42 |  |

*Note*: N=5243. All items were treated as ordinal scales. WLSMV (weighted least squares mean and variance adjusted) was used with GEOMIN (an oblique type) rotation. Comm: communalities. Boldfaced factor loadings are salient (0.30<). *: All factor correlations are significant at least at p<.05. #: Regardless of relevant cross loadings, due to the importance and meaning of this item, it is kept in the factor where it is loaded highest. Analyses are performed with the unweighted dataset. Covariance coverages are between 0.974 and 0.991.

**Supplementary Table 2**. Fit indices of different latent class solution.

|  | **Sample-Size Adjusted BIC** | **Entropy** | **L-M-R test** |
| --- | --- | --- | --- |
| 2 classes | 52443 | 0.974 | 4460.1 (p<.001) |
| 3 classes | 50116 | 0.877 | 2300.9 (p<.001) |
| **4 classes** | **48795** | **0.870** | **1317.4 (p<.001)** |
| 5 classes | 48244 | 0.873 | 564.7 (p=.0605)* |

*Note:* *N*=5237. Lo-Mendell-Rubin adjusted LRT test. *: The solution was not trustworthy due the nonreplicability of the best likelihood value even after the increase of random start in several steps. The accepted solution is boldfaced. Analyses are performed with the unweighted dataset. Covariance coverages are between 0.962 and 0.996.

**Supplementary Table 3.** Sex as a predictor of non-adherence in different age groups

|  | 18-29 years | 30-49 years | 50-64 years | 65≤ |
| --- | --- | --- | --- | --- |
| *Unadjusted* |  |  |  |  |
| Males | 3.35***  [1.90-5.90] | 2.79***  [2.13-3.66] | 3.41***  [2.51-4.64] | 2.00  [1.41-2.84] |
| Females | *Ref.* | *Ref.* | *Ref.* | *Ref.* |
| *Adjusted^#^* |  |  |  |  |
| Males | 3.32***  [1.88-5.87] | 2.82***  [2.14-3.70] | 3.39***  [2.49-4.62] | 2.04***  [1.43-2.91] |
| Females | *Ref.* | *Ref.* | *Ref.* | *Ref.* |

Note: *^#^*: Education and settlement type are controlled. Analyses are performed with the unweighted dataset.
